# Age-Related Changes in Sentence Production Abilities and Their Relation to Working-Memory Capacity: Evidence from a Verb-Final Language

**DOI:** 10.1371/journal.pone.0119424

**Published:** 2015-04-09

**Authors:** Jee Eun Sung

**Affiliations:** Department of Communication Disorders, Ewha Womans University, Seoul, Korea; University of Texas Health Science Center at San Antonio, Research Imaging Institute, UNITED STATES

## Abstract

**Objectives:**

This study investigated the best predictor to capture age-related changes in passive-sentence production using a constrained sentence-production paradigm and explored the role of working-memory capacity in relation to the task demands of the sentence-production tasks.

**Methods:**

A total of 60 participants participated in the study ranging in age from 21 to 86. All were administered a syntactic-priming and a sentence-completion task under either canonical or noncanonical word-order conditions.

**Results:**

Age was significantly and negatively correlated with sentence-production tasks, and the most demanding condition with a noncanonical word order under the syntactic priming paradigm was the best predictor of aging. Working-memory capacity was significantly and positively correlated with all conditions, but the significant correlation remained only for the most demanding condition (the priming task with a noncanonical word order) after controlling for age.

**Discussion:**

Sentence-production abilities were vulnerable to aging, and these effects manifested most clearly when the task demands were high enough to tax individuals’ cognitive capacity. Working-memory capacity partially accounted for age-related changes in sentence-production abilities.

## Introduction

Age-related differences in sentence production and their underlying cognitive mechanisms have received increasing attention. Many researchers suggested sentence-production abilities were vulnerable to aging and elderly adults’ abilities correlated with working-memory (WM) capacity [1]. Researchers defined WM as an underlying cognitive mechanism involved in maintaining and processing linguistic information [2,3]. Evidence suggested that age negatively correlated with performance on WM tasks, suggesting that WM capacity diminishes in elderly adults compared to a young normal group [4–6].

Researchers dedicated a majority of studies on the relationship between WM and language to the language-comprehension modality. Elderly adults with reduced WM capacity showed greater difficulties in sentence-comprehension tasks compared to young normal adults [7–12]. However, relatively few studies directly examined how age-related decline of WM capacity wasrelated to individual differences in sentence-production abilities for elderly adults.

Several researchers reported that older adults produced shorter sentences with simple syntactic structures and delivered less informational content compared to younger adults in spontaneous speech samples [13–15]. However, some limitations exist in analyzing connected language samples to examine syntactic abilities, given that older and even young adults rarely produce complex sentences [16]. Kemper and colleagues [16] employed constrained production tasks to examine abilities of planning and producing complex sentences as a function of linguistic and cognitive demands [13,16–18]. Scholars consistently found that older adults presented diminished performance on controlled-production tasks when task demands were high enough to tax older adults’ limited cognitive capacity.

In the current study, I manipulated task demands in two ways: (a) memory demands, by developing two controlled sentence-production tasks: a sentence-completion task and a syntactic-priming task, and (b) linguistic computational loads by varying the canonicity of a word order in a verb-final language. These sentence-production tasks are novel, given that they are designed to focus only on syntactic features by minimizing semantic-lexical top-down processing in sentence production with a very limited vocabulary used to construct a sentence. I describe specific features of tasks with a novel approach in the section on materials later in this paper. The two tasks share a limited color vocabulary, used to elicit performance on sentence production, but differing in memory demands.

A sentence-completion task is a relatively memory-free task, given that the critical sentential constituents (e.g., nouns and a verb) are written on the picture, and examiners provide the noun phrases to start the sentence corresponding to the picture, which depicts a single action. I designed the other task to demand more memory than the sentence-completion task. A memory-demanding task is based on the syntactic-priming paradigm [19,20], in which people have a tendency to repeat the syntactic structures they hear. During the priming-based production tasks, speakers were asked to produce the target sentence after they listened to the prime sentence. If speakers successfully held the prime sentence in their memory, they were likely to choose the same grammatical structure in a sentence-production task as examiners presented in the prime sentence [19,21,22]. To get the priming effects, the underlying assumption is that speakers successfully hold the prime sentence in memory until they produce the target sentence. Due to the features of maintaining the prime structure in participants’ memory, the priming task is assumed to be a more memory-demanding task than the sentence-completion task, in which relevant information required for sentence construction is already provided.

Another factor I manipulated in the current study was linguistic computational load: canonical vs. noncanonical word order in a verb-final language, Korean. Korean is a predicate-final language with the canonical word order of subject–object–verb. Following the typical word order of subject–object–verb, the subject of a sentence tends to be placed first, but other linguistic constituents can be scrambled freely as long as the predicate comes in the final position [23]. Given that Korean permits variation in the ordering of a verb’s arguments, it is often called a free word-order language, allowing a noncanonical word order of object–subject–verb in Korean [24]. Korean relies on case markers rather than word order to identify grammatical relationships of linguistic elements [25]. Some authors suggested that Korean children are able to use case markers as an index of grammatical function of a word in a sentence to interpret noncanonical sentences such as object–subject–verb by the age of 4 or so [26–28]. In the current study, I manipulated the canonicity of word order based on the assumption that the noncanonical word order might impose greater cognitive demands on sentence production compared to the canonical syntactic structure in Korean passive sentences.

Korean passivization occurs by affixing *i*, *hi*, *li*, or *ki* to verb stems and expressing the by-phrase with a dative case marker *eykey*. Park (2005, p. 195) [29] introduced an example of a Korean active sentence (1) and its passive counterpart (2)(3), shown below, and all Korean examples were transliterated using the Yale system of romanization [30].

1 Active sentence: “Mary caught John.”

**Table:** 

Mary-ka	John-ul	cap-ass-ta.
M-_NOM_	J-_ACC_	catch-_PAST-DECL_

2 Passive sentence (canonical word order): “John was caught by Mary.”

**Table:** 

John-i	Mary-eykey	cap-**hi**-ess-ta.
J-_NOM_	M-_ACC_	catch-_PASS-PAST-DECL_

3 Passive sentence (noncanonical word order): “John was caught by Mary.”

**Table:** 

Mary-eykey	John-i	cap-**hi**-ess-ta.
M-_ACC_	J-_NOM_	catch-_PASS-PAST-DECL_

The subject, Mary, in the active sentence appeared as a *by*-phrase in English with a change of case marker of nominative (*ka*) in the active sentence (1) to a dative form of *eykey* in the passive sentence (2). A passive sentence (2) can be expressed in a noncanonical word order such as a sentence (3), in which accusative word (Mary-*eykey*) was placed as the initial word of the sentence. I examined whether age-related differences exist between canonical and noncanonical structures in passive-sentence production.

Despite the freedom of word order in Korean, noncanonical sentence structures might induce greater difficulties in formulating sentences for elderly adults, based on previous findings in other languages with relatively flexible word order, such as German [31] and Japanese [32]. Researchers consistently suggested that sentences with noncanonical word order (object before subject) were more difficult to process compared to their subject-initial counterparts [31,33,34]. However, previous authors on canonicity effects focused on the comprehension domains in normal young adults, and very few studies investigated the effects of word order on sentence production for elderly adults.

The purpose of the current study was two-fold: (a) to investigate the best predictor to capture age-related changes in passive-sentence production by manipulating task demands, using a constrained production-task paradigm, and (b) to examine whether WM correlates with manipulated task demands manipulated in sentence-production tasks. I manipulated task demands by varying the tasks (a syntactic-priming task and a sentence-completion task) and the word order (canonical and noncanonical). I hypothesized that the syntactic-priming task requires more cognitive resources than the sentence-completion task, which is a relatively memory-free task, and that the noncanonical word order recruits greater linguistic computational resources than the canonical word order, given that participants may need to rearrange the word order when they encounter sentences with a noncanonical word order. I assumed that the best predictor of age would be the sentence-production task with the greatest task demands, such as sentences with noncanonical word order, especially when they were presented in a syntactic-priming task and that WM would be correlated with the task with greater task demands.

## Methods

### Participants

**Fig 1:**
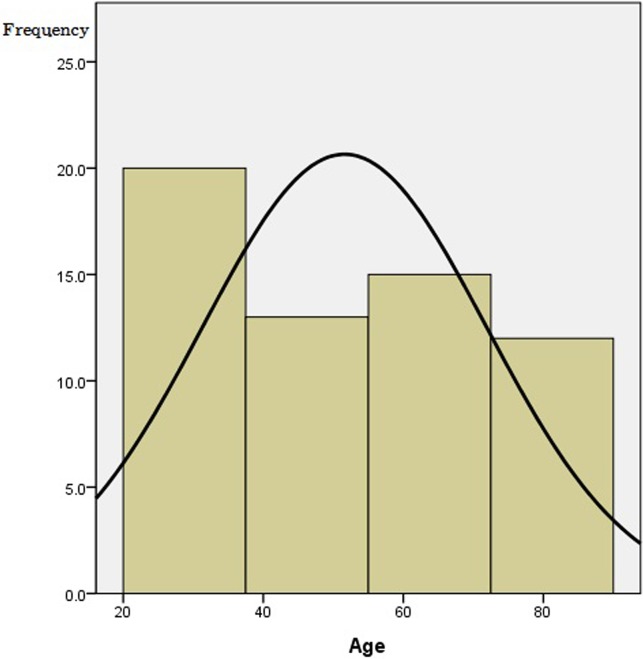
Distribution of age.

A total of 60 individuals (male = 25; female = 35) participated in the study with the age range of 21 to 86. Fig 1 shows a histogram displaying the age distribution from 60 individuals. Their mean age was 52 (*SD* = 20).

All participants provided written informed consent prior to participation. Ewha Womans University’s Institutional Review Board approved the research protocol. None were excluded from the data analysis. All participants were monolingual Koreans, took the Korean Mini-Mental State Examination [35], and showed a normal range of performance on the Korean Mini-Mental State Examination (age- and education-adjusted scores > 16th percentile). Participants had no self-reported history of brain injury, hearing loss, or language developmental disorders.

### Materials

#### Syntactic-priming task

**Fig 2:**
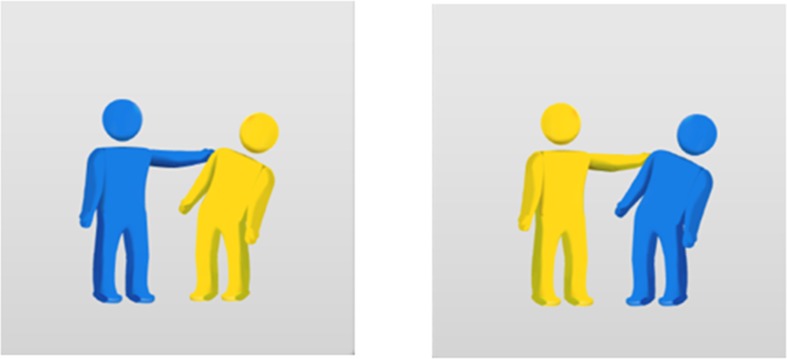
An example of a syntactic priming task.

In the syntactic-priming task, an examiner presented a picture that consisted of two parts: the examiner described the left part of the prime picture, and the right part was the target on which participants were supposed to produce a sentence (Fig 2). Each picture depicted events involved with an agent, a patient, and an action.

Verbs in the sentences were transitive with two-place arguments. To control the top-down lexical-semantic influence on syntactic processing, I created a limited color-related vocabulary with humanized symbols to describe the thematic roles of a sentence. I limited all vocabularies regarding the two arguments (an agent and a patient) to humanized symbols with three different colors: “the Red,” “the Blue,” and “the Yellow.” Examiners provided priming sentences in a full passive sentence. For example, examiners pointed to the left panel of the picture by saying, “the Yellow is pushed by the Blue”; then they asked participants to describe the target picture that depicted that “the Blue is pushed by the Yellow.”

Examiners presented two conditions on the canonicity of the syntactic priming task:

1. A canonical condition in which the noun phrase with a nominative case marker (*ka*) was placed at the head of a sentence followed by a *by*-phrase (*eykey*) (a)
  - Passive-canonical condition

Norangi-ka Parangi-eykey mil-li-ta

The Yellow-_NOM_ The Blue-*by* push-_PASS-IND_

1. Noncanonical condition in which the noun phrase with a dative case marker, *by*-phrase (*eykey*) was placed at the head followed by the noun phrase with a nominative case marker (b).
  - Passive-noncanonical condition

Parangi-eykey Norangi-ka mil-li-ta

The Blue-*by* The Yellow-_NOM_ push-_PASS-IND_

#### Sentence-completion task

**Fig 3:**
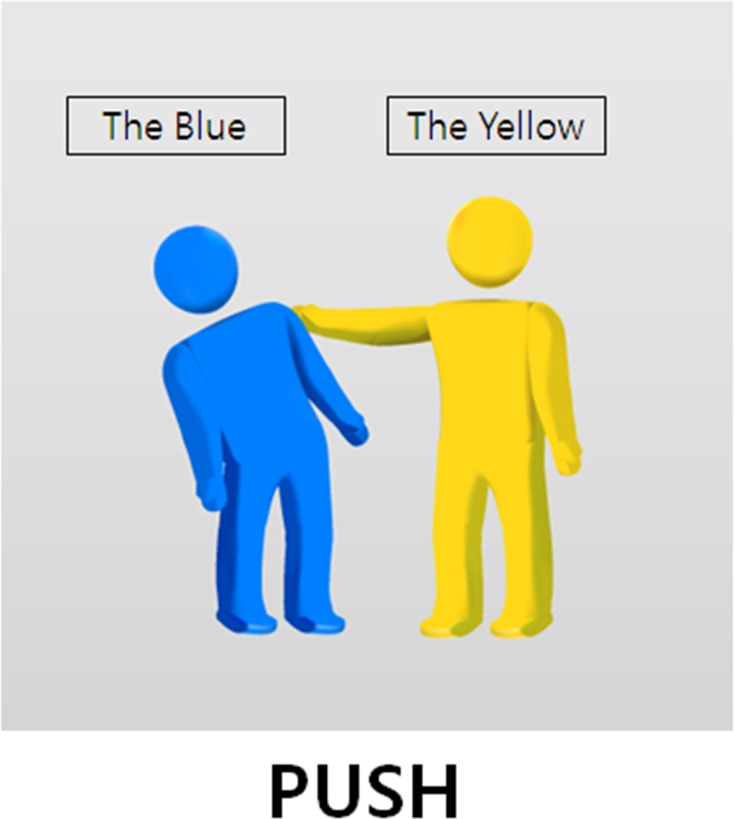
An example of a sentence completion task.

In the sentence-completion task, examiners provided a picture in which the nouns and a verb were written as described in Fig 3. The examiner initiated the first part of the sentence, providing the first noun phrase with either a nominative or dative (*by*-phrase) grammatical marker. Participants were to complete the rest of the sentence to correspond with the picture.

I also presented two conditions of canonicity in the sentence-completion task. In a canonical condition, examiners first initiated the sentence verbally, presenting the noun phrase with a nominative case marker assigned to the patient of a verb such as “Parangi-ka (The Blue-_NOM_),” and asked participants to complete the rest of the sentence. Given that the sentence started with the noun phrase of the patient with a nominative case, participants were forced to use only one option, to use a passive form, to correctly described the picture: “Parangi-ka (The Blue-_NOM_) Norangi-eykey (The Yellow-*by*) mil-li-ta (push-_PASS-IND_).” In a noncanonical condition, examiners provided the head of the sentence with a *by*-phrase and asked participants to complete the sentence saying “Norangi-eykey (The Yellow-*by*) Parangi-ka (The Blue-_NOM_) mil-li-ta (push-_PASS-IND_).”

#### Working-memory capacity measures

Digits-forward (DF), digits-backward (DB), words-forward (WF), and words-backward (WB) tasks served as WM measures. I took the DF and DB tasks from the Korean version of the Wechsler Adult Intelligence Scale (K-WAIS; [36]).The DF task consisted of a total of 14 trials, starting from Span 3 to Span 9 with two trials for each span. When participants failed to recall both trials in a certain span, examiners terminated administration. The total possible score for the task was 14, and the number of correctly recalled trials served as a dependent measure for the task. The DB task had a total of 14 trials, starting from Span 2 to Span 8 with two trials for each span. Sung [37] provided the WF and WB tasks. The procedures and number of trials from word-span tasks were consistent with digit-span tasks. Examiners administered forward-span tasks prior to backward-span tasks, following instructions from the K-WAIS. The order of presentation was counterbalanced between the word- and digit-span tasks.

Prior to the data analyses on WM measures, I conducted exploratory factor analyses using a principal-component-extraction procedure to validate whether the four WM tasks loaded on the same factor. A one-factor solution accounted for 72.61% of the total variance. Based on the results, the average score of the four tasks served as a WM-capacity index for further analyses, as Waters and Caplan [38] suggested that the reliability and stability of WM measures increased when researchers used composite scores.

### Experimental Procedures

Examiners administered a total of 40 stimuli with 20 items for each task type: the syntactic-priming task and sentence-completion task. Each task consisted of canonical and noncanonical conditions with 10 items for each condition. Each task was administered as a block in which canonical and noncanonical sentences were randomly presented. The order of administering the two tasks was counterbalanced across participants to minimize order effects. Responses were coded as correct when participants constructed a passive sentence using correct grammatical markers for each thematic role and correctly inflected verbs. Responses counted as an error when participants did not use the target syntactic structure that examiners primed or initiated with the noun phrase, although they produced grammatically correct sentences. For example, when participants used an active voice to construct a sentence, it was coded as an error because they did not follow the instructions.

## Results

### Correlation and Regression Analyses

To examine whether sentence-production abilities are related to aging, Pearson correlation coefficients were computed among ages and the percentage of accuracy from four types of sentence-production tasks. Age was significantly and negatively correlated with all four conditions of the sentence-production tasks with correlation coefficients of -.50,-.55,-.46, and -.39, (all *p*s < .01) for the priming task with a canonical word order, the priming task with a noncanonical word order, the completion task with a canonical word order, and the completion task with a noncanonical word order, respectively.

To explore the best predictor for age among the four types of sentence-production tasks, a stepwise multiple regression analysis was performed. Age served as a predictor variable. I calculated the percentage of accurate responses for each sentence-production task, and entered the percentages of accurate responses from the four task types (priming-canonical, priming-noncanonical, completion-canonical, completion-noncanonical conditions) as predictors. Results revealed that performance on the syntactic priming task with a noncanonical word order was the only significant predictor for age among the four predictors, *F*(1, 58) = 25.255, *p* <. 0001, *R* ^2^ = .303.

### Partial Correlation Coefficients between Working Memory and Sentence-Production Abilities after Controlling for the Age Factor

Pearson correlation coefficients were calculated among the variables including WM, age, and percentage of accurate responses for each condition of the sentence-production tasks. WM was significantly and negatively correlated with age (*r* = -.77, *p* < .0001). WM was significantly and positively correlated with all conditions of the sentence-production tasks with the correlation coefficients of. 503,. 586,. 474, and. 398, (all *p*s < .01) for the priming task with a canonical word order, the priming task with a noncanonical word order, the completion task with a canonical word order, and the completion task with a noncanonical word order, respectively.

To examine the effects of WM capacity on sentence-production abilities, partial correlation coefficients were computed after controlling for age between WM and each condition of the sentence-production tasks. WM was significantly and positively correlated only with performance on the priming task of the noncanonical conditions, *r* = .296, *p* < .05, after controlling for age. Partial correlation coefficients were *r* = .21, *r* = .23, and *r* = .16, for the priming task with a canonical word order, the completion task with a canonical word order, and the completion task with a noncanonical word order, respectively.

## Discussion

The purpose of the current study was two-fold: (a) to investigate the best predictor to capture age-related changes in passive-sentence production when task demands were systematically manipulated, varying task types (a syntactic-priming task vs. a sentence-completion task) and the canonicity of word order (canonical vs. noncanonical) and (b) to explore the role of WM capacity in relation to the task demands of sentence-production tasks. The study revealed that age was significantly and negatively correlated with all four conditions of the sentence-production tasks with a range of correlation coefficients from-.39 to-.55, indicating that sentence-production abilities decline as one ages. These findings align with previous studies that suggested sentence-production abilities were vulnerable to aging [13,16–18].

The current study employed constrained sentence-production tasks by manipulating task demands, based on the assumption that tasks with greater cognitive demands would be the best predictors of aging. Consistent with the hypothesis, the task with the greatest demands, such as sentences with a noncanonical word order under the syntactic-priming paradigm, was selected as a best predictor of aging. It is interesting to note that canonicity is one of the factors that affected performance on aging-related decline in sentence-production abilities for Korean-speaking individuals, given that Korean is regarded as a relatively free word-order language. Linguistic constituents can be freely scrambled in Korean as long as verbs remain in a sentence-final position. Considering the linguistic features of Korean, canonicity has not gained much attention in Korean sentence-production studies compared to other languages in which the word order is a critical factor that affects performance on sentence production. However, when the canonicity factor was combined with memory-demanding task factors, age-related decline in sentence-production abilities manifested most clearly. The current study suggested that noncanonical word order may be imposed on more linguistic computational loads than canonical word order because participants need to rearrange the word order instead of inhibiting automatically activated canonical word order. To my knowledge, the current study is the first attempt to investigate the effects of canonicity on sentence-production abilities from the perspective of aging. The results are worthy of garnering greater attention to the role of canonicity in Korean sentence-processing studies.

I hypothesized that the syntactic-priming task is a more memory-demanding task than the sentence-completion task, given that participants need to hold the primed syntactic structure in their memory buffer until they produce the target sentence in the syntactic priming task, whereas all linguistic units were provided in the picture they described in the sentence-completion task. Consistent with the hypothesis, the syntactic-priming task, especially with a noncanonical word order, turned out to be the most critical factor that predicted aging-related decline in sentence-production abilities. These results are consistent with previous studies that suggested age-related differences in sentence production were most clearly observed in more complex conditions [5]. The current study suggested that aging effects were maximized when challenging factors were combined.

The current study investigated whether WM capacity affiliates with aging-related decline in sentence-production abilities and how individual differences in WM capacity relate to performance under different conditions from sentence-production tasks, after controlling for age. Results revealed that WM capacity was significantly and negatively correlated with age (*r* = -.77, *p* < .0001) and positively with all conditions in sentence-production tasks. The results suggested that age-related decline in WM capacity may serve as one of the underlying mechanisms that account for diminished performance and age-related changes in sentence-production abilities. Furthermore, WM was significantly correlated only with the condition of a noncanonical word order from the syntactic-priming task when age was controlled. Results indicated that WM might affect individual abilities to produce sentences, and the effects may manifest most clearly in more cognitively demanding conditions. These results are consistent with WM capacity theory [3], which suggested that WM effects emerge most clearly when task demands are high enough to tax individuals’ capacity.

Many studies validated the concept that age-related decline in WM capacity relates to sentence-comprehension ability [8, 39–41]. Previous findings consistently suggested that older adults with reduced WM capacity showed significantly worse performance on sentence-comprehension tasks, especially with complex syntactic structures such as center-embedded sentences. However, few studies directly addressed questions about the relationship between age-related WM decline and sentence-production abilities. The current study provided evidence of the relationship between age-related WM decline and sentence-production abilities.

To summarize, sentence-production abilities were vulnerable to aging, and the age-related decline in sentence-production abilities emerged most clearly in the most WM-demanding tasks. WM capacity partially accounted for aging-related changes in sentence-production abilities. Additional studies are needed to more thoroughly investigate the role of WM capacity in aging-related differences in sentence production, and more various factors need to be explored to most sensitively capture aging-related changes in sentence production. To explore the critical factors and underlying cognitive mechanisms for language is quite critical especially in a rapidly aging society such as Korea, given that this endeavor may contribute to early detection and intervention for aging-related neurodegenerative progress such as mild cognitive impairment and Alzheimer’s disease.

## Supporting Information

S1 Data (XLSX)
